# Farmers’ Perceptions of Dairy Cattle Breeds, Breeding and Feeding Strategies: A Case of Smallholder Dairy Farmers in Western Kenya

**DOI:** 10.1080/00128325.2019.1659215

**Published:** 2019-10-14

**Authors:** M. N. Lukuyu, J. P. Gibson, D. B. Savage, E. J. O. Rao, N. Ndiwa, A. J. Duncan

**Affiliations:** a University of New England, Armidale, Australia; b BEMAR Livestock Consultants, Nairobi, Kenya; c International Livestock Research Institute, Nairobi, Kenya

**Keywords:** breed attributes, dairy cattle, feeding practices, preferences, small-scale farmers

## Abstract

To understand farmers’ preference and perceptions of breed attributes, breeding and feeding practices, 419 households in western Kenya were interviewed in a cross-sectional survey. Respondents scored their preference for cattle breeds, traits and breeding methods on a scale of 1 (most preferred) to 5 (least preferred). Preferences were compared using multinomial logistic regression models on weighted scores. The Ayrshire breed was most preferred followed by the Friesian. Using hardship tolerance as a reference trait, the Friesian was preferred 4.86 times more for high milk production and Ayrshire, Jersey and Guernsey breeds 4.61, 4.60 and 4.18 times (*p *< 0.01) more, respectively, for milk fat content. The Ayrshire was preferred 4.16 times more for its perceived low feed requirement and 1.22 times more (*p *<* *0.01) for resistance to diseases. Friesian was the only breed preferred (3.18 times more) (*p *< 0.01) for high growth rate of calves. Artificial insemination (AI) was the breeding method of choice, but majority (>68%) of respondents used natural mating, because it was readily available and cheaper. The current study highlights the importance of taking into account farmers’ objectives and the production environment when designing breed improvement programmes and recommends packaging of breeding together with feeding interventions.

## Introduction

Dairying, which includes production distribution and sale, offers good opportunities for improving the standard of living of smallholder farmers in low and middle-income countries by the sale of milk, as well as improvement in household nutritional status as a result of higher milk consumption. Local communities benefit indirectly, because dairying provides employment opportunities on the farms and on livestock-related activities, such as feed collection and marketing, as well as veterinary and other services (Baltenweck and Staal 2000). Hence there has been increased adoption of dairy farming and even areas historically dominated by indigenous cattle in Kenya, are gradually being taken over by crossbreds and high grade exotic breeds (Baltenweck and Staal 2000).

Whereas the main reason for keeping cross-bred dairy cows is reported to be their higher milk potential, to the smallholder farmer a dairy cow has a wide range of additional attributes (Baltenweck and Staal 2000; Bebe et al. 2003). Apart from milk, dairy animals (and cattle more generally) also provide manure for the farm, and calves and culled animals for sale. Furthermore, they act as a form of insurance against unforeseen contigencies and are also viewed as a status symbol (Karanja 2003; Ouma et al. 2004b; Moyo and Swanepoel 2010). This broad perspective deviates from many livestock development policies and analyses that place sole emphasis on biological productivity for instnace milk or meat production only, and are therefore at odds with livestock producers’ perceptions and aspirations (Sumberg 2002; Bebe et al. 2003).

Recommendations for smallholder farming systems in East Africa have been to upgrade indigenous cattle to intermediate-grade crosses of small mature size (Rege 1998; Kahi 2002), with a view to retaining adaptive traits, whereas also introducing productive traits in an environment characterised by feed scarcity (Osuji et al. 2005; Lukuyu et al. 2011). However, government practice (by the sale of semen of large dairy breeds) and farmer practice have not followed this recommendation. Apart from the desire to produce more milk, farmers have other objectives, such as producing animals of higher market value and increasing manure production, and have the perception that high-grade cattle can adapt to local feed conditions and diseases (Bebe et al. 2003). Such a divergence in perspective might result in a lack of adoption on the part of producers of what might appear as useful innovations and hamper the formulation of policies that are effective in improving the livelihoods of smallholder farmers (Sumberg 2002; Bebe et al. 2003).

The western Kenya region is a milk deficit area, with most of the milk consumed coming from the Rift Valley Province (Waithaka et al. 2000); hence dairy farming is actively being promoted as a way of improving incomes of the rural communities. Intensification and adoption of superior dairy breeds have been recommended as major strategies for incresing milk production (Musalia et al. 2007). Although the region has favourable climatic conditions for dairying, it is characterized by low milk production and very high levels of poverty (Waithaka et al. 2000), and unlike Central Kenya, association between adoption of dairy technologies and population density has been found to be non-significant (Makokha et al. 2006). Some of the identified constraints to dairy farming in this region are the inefficient breeding system, limited land resources, livestock diseases, poor milk marketing systems and inadequate dissemination of dairy technologies (Mudavadi et al. 2001; Karugia 2012). Cultural beliefs have also reportedly discouraged the uptake of improved dairy breeds (Musalia et al. 2007).

A better understanding of how the multiple functions of dairy cattle can be enhanced in order to fit with farmers’ objectives and the production environment is key to identification of productive and adapted animals for increased milk production without the necessity of increasing the numbers of animals, which might result in land degradation (Philipsson et al. 2011). Because farmers generally adopt and adapt genotypes to their requirements and circumstances (Udo et al. 2011), it is important to know their preference for breed attributes and the breeds they consider most suitable to their circumstances, and how they adapt their management practices, particularly feeding, to match the breeds they keep. Such knowledge can help research and development efforts to deliver the most appropriate genetic, feeding and animal health technologies that match the production environment. The current study investigated the perceptions and preferences of specific attributes and breeds, and the breeding and feeding practices of smallholder farmers in western Kenya in order to understand the rationale underlying their breeding and feeding decisions.

## Materials and methods

### Study sites

The current study was carried out in two sites in the Western Province and one site in the Rift Valley Province in Kenya where a larger project called Dairy Genetics East Africa (DGEA) was on going. The sites were selected according to variability of genotypes, and accessibility. The sites in the Western Province (Butere Subcounty and Kabras Ward) have a high proportion of indigenous and crossbred cattle and a small proportion of high-grade dairy cattle. They lie between the longitudes 34°20′ and 35° E and latitudes 0°15′ and 10° N at an altitude of 1 500 to 1 600 m asl. The average annual rainfall ranges between 1 600 and 2 000 mm yr^−1^, with long rains falling in March to mid-June, and the short rains in mid-August to November. Daily average temperatures range from 18 to 30 °C. This is one of the most densely populated areas in Kenya with density in some parts as high as 1 000 persons km^−2^, and average land holdings of 0.8 ha farm^−1^. The site in Rift Valley Province (Tinderet County, Meteitei Ward) has a high proportion of crossbred and high-grade dairy cattle and a small proportion of indigenous cattle. Tinderet County is situated within the longitude 35°17′ E and latitude 0°30′ N at an altitude of approximately 2 000 m above sea level. The minimum daily temperatures in this region could go to as low as 15 °C during the day and approximately 0 °C during the night, with frost. The area receives an average annual rainfall of 1 900 mm, which is distributed throughout most of the year with a short dry period between December and February (Jaetzold et al. 2005).

### Cross-sectional survey

One circular area with radius 10 km was defined in each of the three study sites. In each area, 31 study locations and 15 reserve locations were randomly selected and defined by GPS points. The closest household to the GPS point was visited and if the household had 2–10 dairy cattle, a trained enumerator used a pretested structured questionnaire to collect data. The respondent was then asked to identify the nearest neighbours keeping dairy cattle. Visits were made to at least five farmers (moving outwards from the GPS point in random directions) who were not more than 0.5 km away and who, i) kept at least 2 dairy animals, ii) when combined with others included a diversity of dairy cattle breeds and iii) preferably had multiple breeds within the household. A total of 419 households (approximately 140 in each site) were visited and each respondent was interviewed once. The survey was conducted during the months of March and April 2011.

Each respondent was asked to list the dairy cattle breeds he or she was familiar with even if they were not present on the farm and to give a preference rank on a scale of 1 to 5 (1 = most preferred, 2 = preferred, 3 = neutral, 4 = not so preferred and 5 = least preferred). For each of the breeds identified, the respondent was asked to list up to three desirable and three undesirable traits and score them as in the case of breeds. Respondents were also asked to list the animal replacement methods available, used and preferred. For the methods used, respondents were asked to score them on a scale of 1 to 5, as with the breeds, and to give the reasons for using those methods (Theis and Grady 1991; ICRA 2007).

Based on the management systems reported in the literature (Mudavadi et al. 2001) respondents were asked to state the management systems they used, the types of feeds fed to different cattle types and the reasons for the practices. Focus group discussions (FGDs) with a subsample of the respondents (in all the sites) were held in order to gain more insight on the perceptions of feeds and feeding practices. Topics explored in the discussions included farmers’ perceptions of the nutritional requirements and status of a cow, feed quality, how they make decisions on types and quantity to feed and the perceived constraints to dairy farming. A checklist was used as a guide and the discussions recorded using a tape recorder, as well as a notebook. Four FGDs were held, with 43 farmers in total (33 males and 10 females).

### Statistical analysis

Frequency counts and means were calculated to obtain number of responses to each of the defined variables and summary statistics presented using cross tabulation. A weighted mean of preference ranking for each trait was calculated using the sum of the product of each rank and its corresponding frequency and dividing by the total number of respondents ranking the specific trait:

$$Y\;=\;\overset{n}{\underset{i\;=\;1}{\sum }}x_{i}f_{i}/N$$

where *Y* refers to the mean weighted rank, $x_{i}\;$is the rank (the ranks, *n* = 5) of the parameter being considered (e.g. breed, trait, breeding method), $f_{i}$ is the frequency of responses for that particular rank and *N* is the total number of respondents ranking the trait.

The relative importance of each breed, associated attribute and breeding methods was calculated using weighted scores. The scores were given in reverse, such that a trait with a preference rank of 1 (highest score) was given a score of 5 (highest). A case of no rank was given the lowest score of 1. The weights were given so that the sum was equal to 1, hence a weight of 5 had a value = 0.335, 4 = 0.268, 3 = 0.200, 2 = 0.130, and 1 = 0.067. The relative importance was then calculated as the sum of the product of the each weighted score and its corresponding frequency and dividing by the total number of respondents involved in the study. Hence for weighted scores using the same formula above, *Y* refers to the importance, $x_{i}\;$is the weight of the parameter being considered (breed, trait, breeding method etc.), $f_{i}$ is the frequency of responses for that particular weight and *N* is the total number of respondents in the study (*N* = 419). The Kruskal–Wallis one-way analysis of variance was used to investigate the differences between scores for the different breeds and breeding methods. As a result of the small number of responses under the Sahiwal, Boran and Zebu breeds, the data were combined and analysed within a single class termed “indigenous breed”, because a sample size with five or less records is too small for the test (Mcdonald 2008). The relationship between the scores and importance given to breeding methods was assessed using correlation analysis.

Preference for traits associated with different cattle breeds was quantified using multinomial logistic regression models. The dependent variables were the array of traits. Hardship tolerance (being able to withstand feed and/or water shortage) was chosen as the reference for the model, because it was the most important trait associated with the indigenous breed from which the dairy animals have been upgraded. Traits such as the ability to produce a considerable amount of milk under conditions of low quantity and quality feeds, coping with unfavourable weather conditions, and the ability to walk long distances in search of feed and water, were classified together as hardship tolerance. An odds ratio of one (1) indicates no difference in preference, more than one indicates higher preference, and less than one indicates lower preference for the specific breed for that particular trait when compared with the indigenous breed. Odds ratios were considered statistically significant if the 95% confidence interval excluded one. Differences in farmers’ perception of feeds supply situation was assessed using log-linear modelling of Likert-type data consisting of responses to nine statements. Responses to each statement consisted of rating from 1 (strongly agree) to 5 (strongly disagree). To additionally investigate the difference between responses from the three survey sites, the data were aggregated to produce “agree” (= strongly agree + agree) and “disagree” (= strongly disagree + disagree) and the neutral response was ignored. Odds ratios for each of the three sites were calculated in Microsoft Excel (2010). All other analyses were performed using Genstat 16 (VSN International 2013).

## Results

### Breed preference

The Friesian and Ayrshire breeds were the dairy cattle breeds mentioned by most respondents (34% each). In addition, they were ranked as most preferred. However, the Ayrshire bred was given a higher (*p *< 0.01) preference ranking than the Friesian breed and it also ranked highest in overall importance, followed by the Friesian breed. The respondents who mentioned the Guernsey and Jersey breeds ranked them as somewhat preferred and they were equal in importance. Generally, very few respondents mentioned indigenous breeds and among those mentioned, the Sahiwal breed was given the highest preference ranking (most preferred) and also scored as the most important among the indigenous breeds. The Zebu was given the lowest preference ranking, but scored equally with the Boran as the least important breed (Table 1).

**Table 1.** Number and percentage (%) of respondents mentioning various cattle breeds, their mean preference rankings and importance in cross-sectional survey of 419 smallholder farms in western Kenya.

Breed	Number of respondents mentioning	Percentage of total Respondents (%)	Mean preference ranking^1^	Importance^2^
Friesian	362	34.3	1.6	4.3
Ayrshire	360	34.1	1.3	4.6
Jersey	123	11.7	2.1	2.8
Guernsey	146	13.8	2.1	2.9
Sahiwal	26	2.5	1.8	1.6
Boran	16	1.5	2.8	1.2
Zebu	23	2.2	3.2	1.2

NB: Ranking of more than one breed by one respondent was possible

^1^Preference ranking: (five-point scale where 1 = most preferred and 5 = least preferred); ^2^Importance was calculated using a weighted mean of all scores (5 to 1) of a particular breed (including no score given a default weight of 1): 5 = most important, 4 = somewhat important, 3 = neutral, 2 = not so important, 1 = least important.

### Preferred traits

In general, the traits of importance in their order of priority (from most important) were milk production, milk fat content, resistance to diseases, low feed requirement, hardship tolerance and high growth rate of calves. Market value and fertility were ranked as the least important traits. When it came to traits associated with specific breeds, the Friesian was considered outstanding for milk production and Ayrshire for milk fat content. The Ayrshire breed also scored highly in importance, because of perceived resistance to diseases and low feed requirement. Milk fat content was important in the Jersey and Guernsey breeds, but generally all other traits were of low importance. The most important traits for the indigenous breeds were high hardship tolerance and disease resistance (Table 2).

**Table 2.** Importance of desirable cattle traits in specific breeds in a cross-sectional survey of 419 smallholder farms in western Kenya.

		Importance^1^ of specific breeds				
Trait	Overall general importance	Friesian	Ayrshire	Jersey	Guernsey	Indigenous
Milk production	4.7	4.5	2.6	1.5	1.5	1.2
Milk BF content	4.4	1.4	3.8	2.6	2.5	1.1
Market value	1.5	1.1	1.0	1.1	1.1	1.2
Coat colour	1.7	1.2	1.4	1.1	1.1	1.0
Resistance to diseases	3.1	1.3	2.5	1.3	1.3	1.7
Growth rate of calves	1.7	1.5	1.2	1.0	1.0	1.0
Feed intake	3.0	1.2	2.5	1.5	1.5	1.1
Fertility (CI)	1.4	1.2	1.0	1.0	1.0	1.0
Others^2^	2.7	1.3	1.7	1.1	1.3	1.8

^1^The importance was calculated using a weighted mean of scores for all rankings of a particular trait against each breed (including no ranking given a default score of 1); 5 = most important, 4 = somewhat important, 3 = neutral, 2 = not so important, 1 = not important at all. ^2^Most important was hardship tolerance. Others were attractive looks, quick recovery from disease, temperament and body size. BF = Butter Fat; CI = Calving Interval

When compared with indigenous breeds, farmers preferred Friesians for their high milk production (4.86 times greater than for hardiness) and for Ayrshire, Jersey and Guernsey breeds, preference was related to milk fat content (4.61, 4.60 and 4.18 times more than hardiness, respectively). The Ayrshire breed was most highly preferred for low feed requirement compared with hardship tolerance (4.16 times more) and was the only breed preferred for resistance to diseases (1.22 times more compared with hardiness). The Friesian was the only breed preferred for high growth rate of calves (3.18 times more compared with hardiness) (*p *< 0.01). Market value, coat colour and fertility did not influence farmers’ preference for any breed (Table 3).

**Table 3.** Odd ratios (and their 95% confidence intervals) from multinomial logistic regression for the preferred traits of dairy cattle breeds in cross-sectional survey of 419 smallholder farmers in western Kenya.

Preferred trait^1^	Friesian		Ayrshire		Jersey		Guernsey	
Hardship tolerance	ref.		ref.		ref.		ref.	
Milk production	4.86	(3.79, 5.92)	2.42	(1.45, 3.38)	1.98	(0.78, 3.17)	1.50	(0.37, 2.64)
Milk BF content	2.83	(1.12, 4.45)	4.61	(3.11, 6.12)	4.60	(2.99, 6.22)	4.18	(2.61, 5.74)
Market value	0.59	(ns)*	−1.36	(ns)	−0.39	(ns)	0.30	(ns)
Coat colour	17.33	(ns)	16.97	(ns)	16.98	(ns)	16.75	(ns)
Resistance to diseases	−0.05	(ns)	1.22	(0.44, 2.00)	0.35	(ns)	0.35	(ns)
Growth rate of calves	3.18	(1.53, 4.83)	1.17	(ns)	0.52	(ns)	0.12	(ns)
Feed requirements	3.04	(0.82, 5.23) ns	4.16	(2.10, 6.22)	3.70	(1.51, 5.89)	3.45	(1.31, 5.59)
Fertility (CI)	18.06	(ns)	17.39	(ns)	1.76	(ns)	1.46	(ns)

*ns = not significant (odds ratio was significant, if the 95% confidence interval excluded one), means the respondents did not consider the trait as important. ^1^Preference for a breed for each trait was compared with preference for hardship tolerance

### Breeding methods

Natural mating to local bulls was used by the majority of respondents (89%), because of its relatively low cost and high availability. Most of the bulls used were privately owned and were rated higher in preference rankings compared with community-owned bulls. Nearly three times as many farmers had access to and used neighbours’ bulls than had access to and used their own bulls. Although artificial insemination (AI) was the most preferred (*p* < 0.01) breeding method, it was accessible to only 42% of the respondents and used by only 28%. Although private AI services, compared with government AI services, were used by a larger proportion (28% versus 6%) of respondents, because it was more readily available, a government AI service was more preferred. Access to and use of cooperative and NGO AI services, as well as the use of imported semen, was generally low. The average cost of AI services by all the service providers (government, private, NGO and dairy cooperative) was similar; however the cost of private AI services was highly variable (mean = 1 314; SD = 1 063.9), whereas the cost a cooperative AI service was generally fixed (SD = 0.0). However, there was only one dairy cooperative society in the area and most of its activities were dormant. Local semen (not imported) from the cooperative was slightly more preferred than semen from all other sources although in general, preference for the various sources was not significantly different (Table 4). Cost, availability and the necessity to acquire better quality breeds with the desired traits were the main factors that influenced choice of breeding service.

**Table 4.** Availability, use and preference ranking of breeding methods in a cross-sectional survey of 419 smallholder farms in western Kenya.

Breeding method^#^	Number with access	Number using	Mean rank^1^ ± SE	Importance^2^	Average cost^3^ (KES)
Own bull	103	90	1.7 ± 0.02	2.9	600 (396.4)
Other bull	337	284	2.1 ± 0.12	3.8	394 (203.9)
Community bull	15	8	2.4 ± 0.02	1.2	417 (195.1)
AI local/ government	64	25	1.4 ± 0.02	1.8	1 278 (361.4)
AI local/ private	177	118	1.5 ± 0.02	3.3	1 314 (1 063.9)
AI local/ cooperative	9	3	1.3 ± 0.01	1.1	1 200 (0.0)
AI local/ NGO	20	5	2.5 ± 0.03	1.1	1 220 (560.0)
AI imported/ government	20	5	1.4 ± 0.02	1.2	1 750 (531.5)
AI imported/ private	41	18	1.4 ± 0.02	1.6	2 553 (1 211.9)
AI imported/ cooperative	1	1	1.0 ± 0.00	1.0	6 400 (0.0)
AI imported/ NGO	5	2	1.0 ± 0.00	1.1	4 450 (1 950.0)

^#^Use of more than one breeding method by one respondent was possible. AI = artificial insemination.

^1^Preference ranking: 1 = Most preferred; 2 = Preferred; 3 = Neutral; 4 = Not so preferred; 5 = Least preferred). Sample size less than 5 was excluded from the Krusal–Wallis one-way analysis of variance; ^2^Importance was calculated using the average weight of scores of a particular method (including no score given a default weight of 1); ^3^Values in parenthesis are the standard deviation

Only 18% of the respondents purchased replacement animals. Of these 53% purchased from markets outside the locality and only 14% purchased from large-scale farms. The average cost of animals was KES 16 000 in the local markets and in neighbouring farms, KES 25 000 in markets outside the locality and KES 55 500 (but could be up to KES 100 000) from large-scale commercial dairy farms.

### Management systems

The majority of respondents (96.9% of HH) had crossbred cattle, with approximately 61% practicing free grazing. Only 24% of the households keeping crossbred cattle practiced pure zero grazing and mainly for the mature animals; the majority of respondents kept weaners and calves under free a free grazing system. Indigenous breeds were found in approximately 24% of the households and most of them (approximately 80%) were kept under the free grazing system (Table 5).

**Table 5.** Number of households keeping various cattle types and the percentage (%) practicing different management systems in a cross-sectional survey of 419 smallholder farms in western Kenya.

		Cross and pure breed		
Management system	Indigenous breed	Mature cattle	Weaners	Calves
Free grazing	82 (79.6)	252 (60.5)	119 (84.4)	223 (67.0)
Semi-zero grazing	14 (13.6)	65 (15.5)	15 (10.6)	53 (15.9)
Zero grazing	7 (6.8)	89 (24.0)	7 (5.0)	61 (18.3)
Total HH with cattle type*	103	406	141	333
Average number HH^−1^	1.66	2.41	1.20	1.42

* One household (HH) can have more than one cattle type and practice more than one management system; numbers in parentheses represent the percentage of total HH with that cattle type

### Feeds and feeding

Approximately 47% of farms depended on unimproved natural pastures as their main feed resource. Unimproved natural pastures comprised a mixture of grasses, the common ones being Kikuyu grass (*Pennisetum clandestinum*), star grass (*Cynodon* spp.) and Napier grass (*Pennisetum purpureum*). Approximately 51% of the respondents had planted on average 0.5 ha of Napier grass on their farms. Crop residues particularly maize stover (*Zea mays*) was an important feed resource particularly in the zero-grazing system and it was mostly available on-farm, because maize was a major food crop to 94% of the households. Approximately a quarter (23%) of the respondents reported they had planted fodder trees mainly calliandra, (*Calliandra calothyrsus*) or herbaceous legumes to feed to lactating or in-calf cows, but only six households had 100 or more calliandra trees. Some 5% of the respondents had planted desmodium (*Desmodium intortum*), but the average quantity was 0.3 ha. Approximately half (52%) of the respondents reported that they purchased fodder, mostly Napier grass, from other farms and natural cut grass harvested from public land, but only 12% had purchased any fodder during the previous year. The majority of these had purchased Napier grass (6%) or cut grass (4%). Almost all (96.7%) the respondents reported that they fed a certain supplement to their cattle, but only commercial dairy meal and minerals were fed by a relatively high proportion of respondents (53.5 and 59.9%, respectively). Generally, supplements were fed mainly to lactating cows (Table 6).

**Table 6.** Common feed resources in households involved in a cross-sectional survey of 419 smallholder farms in western Kenya.

Fodder/feed type^1^	Number of respondents using feed type	Percentage of total respondents	Average quantity planted/ purchased among those using	Average quantity planted/ purchased among all households	Units
On-farm basal					
Natural unimproved pasture	167	39.9	1.8	0.72	ha
Napier grass	214	51.1	0.5	0.23	ha
Planted pasture	9	2.1	0.4	0.008	ha
Desmodium	20	4.8	0.3	0.006	ha
Maize^2^	394	94.0	1.0	0.9	ha
Calliandra	58	13.8	48	3.9	trees
Luceana	9	2.1	36	0.4	trees
Sesbania	7	1.7	19	0.2	trees
Off-farm basal					
Napier grass	24	5.7	3.6	0.16	ha yr^−1^
Cut grass	16	3.8	11.3	0.3	sacks^3^ yr^−1^
Commercial supplements					
Dairy meal	245	58.5	1.9	0.97	kg cow^−1^ day^−1^
Commercial mineral salt	382	91.2	10	10	g cow^−1^ day^−1^

^1^One household can use more than one fodder/feed type; ^2^Maize was a source of crop residues; ^3^One standard sack of cut grass contains approximately 15–20 kg of fodder.

### Farmers’ perceptions of feeds and feeding

The majority of farmers (68.5%) agreed that availability of fodder on-farm resulted in increased milk production, but disagreed there were enough or different varieties of feeds available on-farm (50.6% and 64.6%, respectively). There was a lack of consensus on most of the other feed supply situations (access to off-farm feeds, affordability and effect on milk production, and access to forage seeds) with equal proportion of total number of farmers somewhat agreeing or disagreeing. The proportion of farmers strongly agreeing or disagreeing with the various options for feed supply situations was generally low. There was a strong interaction (*p *< 0.01) between sites and the responses. Although the level of agreement with each situation varied, farmers in Butere generally were most likely to agree with the feed supply situations, whereas those in Mateitei were most likely to disagree. Farmers in Kabras were slightly in agreement (Table 7).

**Table 7.** Farmers’ perceptions of feed supply situation based on number of respondents agreeing or disagreeing with various options in a cross-sectional survey of 419 smallholder farms in western Kenya.

	Butere			Kabras			Meteitei			*p*
Feed supply situation	Agree	Disagree	Odds ratio	Agree	Disagree	Odds ratio	Agree	Disagree	Odds ratio	
There is sufficient on-farm feeds all year round	76	44	2.71	71	70	1.29	36	98	0.29	<0.001
Different types of feeds are available on-farm	86	40	25.57	15	93	0.31	4	133	0.04	<0.001
Availability of on-farm feeds can result in increased milk production	109	9	6.26	126	11	6.40	52	81	0.05	<0.001
Off-farm feeds are easily accessible	105	9	27.02	59	58	1.06	17	118	0.06	<0.001
Different types of feeds are available off-farm	103	10	53.61	26	82	0.36	13	121	0.08	<0.001
Availability of off-farm feeds can result in increased milk production	85	27	4.78	66	22	3.89	23	113	0.07	<0.001
Off-farm feeds are affordable	91	10	19.20	55	43	1.42	18	111	0.06	<0.001
Farmers have sufficient information on feeding dairy animals	11	5	47.49	38	55	0.50	34	99	0.14	<0.001
Forage seeds are easily accessible	94	31	6.98	39	69	0.57	34	99	0.26	<0.001
*n*	134			147			138			

Note: Agree = strongly agree and somewhat agree; Disagree = somewhat disagree and strongly disagree; Neutral and no answer were excluded

The farmers were aware of the general requirements of a dairy cow, the major ones being improved health care and feeding sufficient basal feed. However there was consensus that insufficient feed supply and disease incidence were major challenges faced by the farmers. High cost of inputs and services was the biggest challenge, consequently the farmers were not able to meet their animals’ requirements, because of lack of funds (Table 8).

**Table 8.** Smallholder farmers’ perceptions of factors associated with specific aspects of dairy management discussed in focus groups in western Kenya**.**

Management issue^1^	Associated factor	Number of mentions*
General requirements	Improved health care	18
	Feeding of sufficient basal feeds	13
	Feeding of supplements	6
	Improved general Welfare	2
	Provision of water	3
		
Determination of feed offered	Milk yield	13
	Availability	12
	Breed	6
	Body size	2
	Information from consultations	4
	Age	1
	
		
Constraints to dairying	High cost of inputs and services	8
	Animal diseases	6
	Insufficient feed	6
	Lack of information	4
	Lack of market for milk	2
	

*The number of mentions was used as an indicator of the relative importance of the factor. **^1^**Mention of more than one factor per focus group discussion was possible. Questions used to address specific management issues:

i. What are the general requirements of a dairy cow in order to support high milk production?

ii. Which factors should be considered when making decisions on the quantity of feed to be offered to dairy cattle?

iii. What are the major constraints that farmers face in smallholder dairy farming?

## Discussion

Milk production was the most important trait that farmers considered when selecting breeds and this was confirmed by the high ranking and dominance of the Friesian and Ayrshire breeds (34% of the farms) compared with Jersey and Guernsey (approximately 12% of the farms). This is not surprising, because milk production for feeding the family and for generating cash income had previously been cited as the main objective for keeping cattle among smallholder farmers in Kenya particularly in areas where dairy farming is actively promoted (Makokha et al. 2006). The high preference for Friesian and Ayrshire breeds could also signify the perception that animals of large body size produce more milk. However, this perception neither translated into any detectable strategies for increased on-farm forage production, which is critical to meeting the increased feed requirement of high producing animals, nor feeding strategies for meeting individual animals’ feed requirements. Instead, as is common, among smallholder farmers, the practice was to offer basal diet to all the animals as a group and then feed supplements (in most cases 2 kg dairy meal day^−1^) (Kaitho et al. 2001; Njarui et al. 2011a) to the lactating animals during milking. Whereas this strategy might not have observable impact on milk yield, it could indicate that farmers are aware that supplementary feeding can result in an increase in milk yield.

The farmers in the current study were aware that large animals consume more feed, but the amount offered was largely determined by availability. Studies conducted in smallholder farms show that planning for feed supply is not common and instead, farmers use small quantities of whatever feed is available and rarely if ever is there an excess, hence feed supply varies on a daily, as well as on a seasonal basis (Methu et al. 2000; Romney et al. 2005; Lanyasunya et al. 2006). Additional upgrading of dairy herds in smallholder farms involved in the current study might not result in the expected improvement in milk production unless strategies for improved feeding are put in place.

Despite scoring lower than Friesian on milk production, the Ayrshire scored highest on overall importance, as a result of perceived high milk fat content, low feed requirement, resistance to diseases and hardship tolerance. This confirms that farmers are interested in more than one trait when selecting breeds and they were willing to forego very high milk production and select a breed perceived to be more resilient. This has implications for breeding programmes targeted at smallholder farmers. Preference for a balance across multiple traits is common among farmers keeping various livestock breeds and even species, particularly in developing countries (Mwacharo and Drucker 2005; Ouma et al. 2007). Hence, breed improvement programmes should not focus on a single trait, such as milk production, ignoring adaptive traits or the multiple roles played by cattle in most rural communities, because this could result in genotypes that do not meet the requirements of the farmers (Ouma et al. 2004a).

The high importance attached to milk fat content is most likely associated with consumer preference (perceiving milk with higher, butterfat content as tastier) rather than the market, because milk payment is based on volume rather than quality. Milk consumers especially in rural areas have been shown to prefer unprocessed milk, because of the lower cost and better taste, as a result of higher milk fat content (SDP 2004; Njarui et al. 2011b). In the current study, the perception of high disease resistance in the Ayrshire could be attributed to the relatively low experience with other breeds for instance, Jersey and Guernsey and hence the only comparison was with the Friesian. However, the considerable number of farmers who were familiar with Jersey and Guernsey breeds (12 and 14%, respectively) could be an indication that there is an opportunity for promoting them as alternative breeds. In the current study, indigenous breeds were not given high importance probably, because the respondents (who were mainly dairy farmers) could have already been in the process of upgrading their existing herds.

Farmers ranked the Friesian breed high for fast growth rate of calves, which can be of advantage to farmers aiming at producing heifers for sale. However, market value was not rated an important trait in the current study and hence the preference for higher bodyweight and high growth rate of calves might be related to early breeding of heifers, because the farmers are still in the process of building their dairy herds.

The surprisingly low importance given to fertility could indicate farmers’ lack of knowledge of the relationship between reproductive performance and milk production. It might also be plausible that farmers tended to associate breeding success with the breeding service, rather than the animal. Success rate has been given as one of the factors influencing choice of breeding service (Baltenweck et al. 2004; Murage and Ilatsia 2011). In the current study, however, only a small proportion (2%) of farmers mentioned success rate as a reason for choice of breeding method, which might also indicate a knowledge gap. This might be attributable to the fact that the area is still in the early stages of dairy development (Mudavadi et al. 2001; Musalia et al. 2007). The low preference for fertility traits might also reflect that fertility is not as important in these systems as external experts tend to think it is.

Crossbreeding offers one of the most efficient and quickest ways of improving the productivity of dairy herds; hence access to high quality germplasm is key to sustained dairy development. In the current study, natural mating was the most accessible and widely used breeding method although artificial insemination more preferred. This might be an indication that farmers are constrained in their choice of breeding service (Baltenweck et al. 2004; Mugisha et al. 2014). Poor access to Artificial Insemination (AI) is a common situation among smallholder farmers, as a result of privatization of the service in the early 1990s, which resulted in emergence of private AI service providers. Private AI services are relatively too costly for most farmers (Omiti 2002; Murage et al. 2006). Indeed, farmers in the current study identified high cost and unavailability of an AI service as the main reason for using natural service. As a result of the business orientation, private AI service providers tend to be concentrated in areas with high densities of dairy cattle where the demand for the service is high and the returns from milk are able to support its use. The relative scarcity of private AI service providers could be attributed to the low density of dairy cattle in the study area. Although the cost of AI by the cooperative was invariant, only a small number of farmers reported the service as accessible. This was most likely from their experience in the past, because the dairy cooperative in this area was not functional (Mudavadi et al. 2001). It is often speculated that use of bulls could result in inbreeding and retrogression, because most of these bulls are of unknown pedigree. Furthermore, it was found that only 40% of farmers could give an accurate estimation of their animals’ breed composition (Weerasinghe et al. 2013) hence it might be difficult to define a breeding program. However, the level of inbreeding in cattle owned by smallholder farmers in Kenya has been found to be low (Anunda 2010; JP Gibson and O Mwai, International Livestock Research Insititute, Nairobi, Kenya, pers. comm.). In areas where farmers are constrained by unavailability of AI services, use of bulls could be an option, but it must be supported by an efficient recording system, both at farm and community/government level so that the pedigree of the bull is known. Record would also play a big role in curbing inbreeding.

The fact that very few respondents (18%) purchased dairy cattle for replacement indicates that the large majority of farmers rear their own replacements (even farmers who purchase replacements could also be rearing their own replacements). Furthermore, the majority of farmers purchased animals from the markets, most likely because of the high cost of animals coming from the large-scale farms. Few farmers can afford to buy animals at a price more than KES 30 000 (approximately USD 300) (Musalia et al. 2007), as charged by large farms. Replacement animals purchased from markets do not have production or pedigree records and their genetic merit cannot be ascertained. This situation portrays the dire necessity for an AI service, if these farmers are to successfully upgrade their animals.

In the current study, farmers’ perception of feed supply situations and breed preference were not correlated. This could indicate that farmers do not consider the breed of animals when making feeding and other management decisions. In contrast with earlier studies, the main reason given for adoption of zero grazing was disease prevention rather than diminishing land size, despite the area having a high human population density and low average land size (Jaetzold et al. 2005). The low level of intensification in western Kenya could be attributed to poor access to input and output markets particularly breeding and extension services and lack of an efficient milk marketing system (Mudavadi et al. 2001; Karugia 2012). Lack of credit to allow for investment in technological changes is another constraint to intensification among smallholder dairy farmers (Romney et al. 2000).

Natural unimproved pasture, Napier grass and crop residues formed the highest proportion of feed resources. This is common amongst smallholder dairy farmers in Kenya (Katiku et al. 2011; Lukuyu et al. 2011). Although respondents in Butere agreed that there was enough on-farm feed throughout the year, participants of the focus group discussions were aware that on-farm resources were not enough to meet the animals’ requirements. This apparent contradiction could indicate a lack of knowledge of the animals’ requirements. The majority of farmers who practiced zero grazing were in Butere. Feed supply is a major activity intensive dairy systems and this could explain why these farmers were more likely to agree with the various options of feed supply situations. Although the respondents agreed that fodder was available off farm, it is unlikely that purchased fodder would be able to meet the deficit in the area of study, because this is only feasible where smallholder dairy is highly developed and the returns from milk are able to meet the cost of external inputs.

Herbaceous legumes have shown potential to increase milk production, but very few farmers had planted them. Lack of planting material, poor establishment and lack of persistence have been found to limit incorporation of these supplementary fodders into the farming systems and their utilisation as feeds (Mureithi et al. 1998; Mwangi and Wambugu 2003). Indeed, most respondents disagreed that fodder seeds were easily accessible. In addition to the aforementioned factors, competition for land with food crops and lack of information could be other factors associated with the low adoption. Although calliandra has been shown to be a suitable replacement for the commercial dairy meal, the amounts reported on farms in the current study were too low to impact on productivity. In Kenya, the recommendation to farmers is that 500 plants, managed in a hedgerow, can provide enough leaf annually to supplement the diet of one dairy cow (Franzel et al. 2014). Because a substantial proportion of respondents (53%) fed dairy meal, there could be a high probability of adoption of calliandra among dairy farmers in the study area, if an effective extension and plant supply program was implemented. In addition, the fact that leguminous trees have additional benefits of adding nitrogen in the soil makes calliandra potentially important for increasing feed supply and improving soil fertility. Generally, use of concentrates in smallholder farms is low, with the majority feeding a flat rate of 2 kg day^−1^ throughout the entire lactation (Kaitho et al. 2001). Access to credit facility and an efficient milk marketing system have been shown to significantly increase the use of concentrate and hence milk yield (Romney et al. 2000). Strengthening of dairy cooperatives can be an effective strategy towards improving access to credit and other services by smallholder dairy farmers.

## Conclusion

The Ayrshire was perceived as the most important breed, thanks to its productive traits (milk yield and milk, butter fat content), as well as adaptive traits. The respondents demonstrated awareness of serious feed constraints by preferring dairy cattle breeds with low feed requirement. However, there was an apparent contradiction in their preference for animals of large body size with high genetic merit for milk production. A disconnect between breeding and feeding strategies was evident, because farmers exhibited a lack of knowledge of basic principles of feeding for milk production. The farmers involved in the current study were most likely in a transitional stage of intensification, as indicated by their preference for larger dairy breeds probably driven by the requirement that surplus marketable milk should earn income, and relatively low adoption of the zero-grazing system of management. Constraint in the choice of breeding method was evident, because respondents preferred artificial insemination (AI), but the majority used bull service, because it was cheap and readily available.

## Recommendations

- Dairy development strategies for smallholder farmers, such as those involved in the current study, should adopt a multipronged approached, comprising a well-designed and implemented breeding programme and an effective extension and marketing support system.
- For sustainable dairy development, the production circumstances of the farmers should be carefully considered and the capacity of farmers to adopt and adapt technologies to their systems harnessed.
- In order for farmers to obtain the benefits of upgrading their indigenous breeds to high levels of exotic genes, breeding interventions should go hand in hand with feeding interventions.
